# Outcome of Sleeve Gastrectomy Converted to Roux-en-Y Gastric Bypass and One-Anastomosis Gastric Bypass

**DOI:** 10.1007/s11695-021-05866-0

**Published:** 2022-01-14

**Authors:** D. M. Felsenreich, K. Steinlechner, F. B. Langer, N. Vock, J. Eichelter, C. Bichler, J. Jedamzik, M. Mairinger, I. Kristo, G. Prager

**Affiliations:** 1 Division of Visceral Surgery, Department of General Surgery, Medical University of Vienna, Vienna, Austria; 2 Bariatric and Metabolic Surgery, Department of General Surgery, Medical University of Vienna, Waehringer Guertel 18-20, 1090 Vienna, Austria

**Keywords:** Sleeve gastrectomy, GERD, Weight regain, Conversion, Quality of life, Roux-en-Y gastric bypass, One-anastomosis gastric bypass

## Abstract

**Purpose:**

Sleeve gastrectomy (SG) is the commonest bariatric procedure worldwide but there is also a high conversion rate mainly due to weight regain and gastroesophageal reflux disease (GERD) reported in studies with long-term follow-up. The aim of this study is to highlight benefits and limitations of converting SG patients to Roux-en-Y gastric bypass (RYGB) and one-anastomosis gastric bypass (OAGB).

**Setting:**

Retrospective cross-sectional-study, medical university clinic setting.

**Methods:**

This study includes all patients converted from primary SG to RYGB or OAGB by 12/2018 at the Medical University of Vienna. Patients were examined using gastroscopy, esophageal manometry, 24-h pH-metry, and questionnaires.

**Results:**

Fifty-eight patients were converted from SG to RYGB (*n* = 45) or OAGB (*n* = 13). Total weight loss of patients converted to RYGB and OAGB was 41.5% and 44.8%, respectively, at nadir. Six patients had Barrett’s esophagus (BE) after SG. In four out of these six patients, a complete remission of BE after conversion to RYGB was observed; nevertheless, two patients after RYGB and one after OABG newly developed BE. Clinical GERD improved at a higher rate after RYGB than after OAGB. Both revisional procedures improved associated medical problems.

**Conclusion:**

Conversion to RYGB is probably the best option for patients with GERD after SG. OAGB has shown a low potential to cure patients from GERD symptoms after SG. In terms of additional weight loss and remission of associated medical problems, both procedures studied were equal. Surveillance gastroscopies every 5 years after SG revisions are recommended.

**Graphical abstract:**

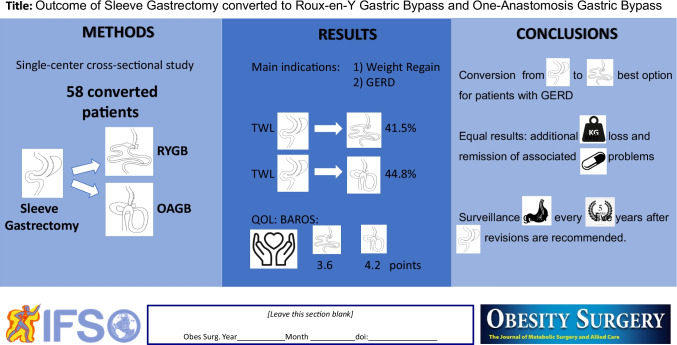

## Background

The numbers of bariatric and metabolic operations constantly increase every year, which holds true not only for primary procedures but also for revisional surgeries as well. It becomes more and more generally accepted that morbid obesity is a chronic recurrent disease that may not be cured by a single surgical procedure in some cases [1].

Laparoscopic sleeve gastrectomy (SG) is the most commonly performed bariatric procedure worldwide [2] but studies with long-term follow-up also report a high conversion rate [3, 4]. Causes for conversions and revisional procedures are manifold, for instance, weight regain, insufficient weight loss, symptomatic gastroesophageal reflux disease (GERD), or acute issues after the SG [3, 5]. Re-operative procedures offered to SG patients can vary, as patients suffering from GERD may benefit most from a conversion to Roux-en-Y gastric bypass (RYGB) [6], while patients with weight regain or insufficient weight loss may profit from a more malabsorptive procedure such as one-anastomosis gastric bypass (OAGB) or single-anastomosis duodeno-ileal bypass + sleeve gastrectomy (SADI-S) [7, 8]. Further options are laparoscopic re-sleeve [9] or the use of endoscopic suturing devices [10] to re-increase the restriction of the sleeve, which will not be addressed in any detail in this paper, as these procedures were not performed in the present cohort.

The aim of this study is to highlight the benefits and limitations of converting SG patients to other bariatric procedures based on the results of objective examinations such as gastroscopy, esophageal manometry, 24-h pH-metry, bariatric outcome scores, and standardized quality of life (QOL) questionnaires.

## Patients and Methods

All patients that were converted from SG to other bariatric procedures between January 2003 and December 2018 at the Medical University of Vienna were analyzed for this single-center study, irrespective of the bariatric center having performed the primary SG. Conversions from SG to RYGB, OAGB, and SADI-S were identified. Nine patients with gastric banding, two with fundoplication, and one with gastric stimulation before the SG were identified. These 12 revisional patients were not included in the further analysis as their increased risk of gastroparesis may have affected the results. Additionally, patients converted from SG to SADI-S were excluded from further analysis as this group was very small (*n* = 8) with a follow-up period too short to be able to draw any general conclusions.

All included patients were invited to go through the following examinations: gastroscopy; esophageal manometry; 24-h pH-metry; bariatric outcome scores; standardized QOL questionnaires; and interviews about their conversion, history of weight, symptoms, and potential complications.

This study has been approved by the Ethics Committee of the Medical University of Vienna as the local institutional review board *(reference number 1894/2019*).

### History of Weight and Associated Medical Problems

Patients’ history of weight was evaluated by interviewing, operation reports, and records of previous weighing. Thus, the following information was gathered: weight at the time of the SG and the conversion, lowest postoperative weight achieved after SG and the conversion, as well as the current weight.

Associated medical problems (AMP) and reflux were documented and recorded at the time of the SG, at the time of the conversion, and at the time of the follow-up visit for the two groups (RYGB and OAGB).

### Conversion

Data including reasons for the conversion, symptoms, complications, and the date of the conversion were collected. The patients were grouped by the procedure they were converted to (RYGY and OAGB).

In all patients converted to RYGB or OAGB, the pouch was resized using a 12-mm Bougie. The limb lengths in RYGB were created as follows: 70 cm biliopancreatic limb (BPL) and 150 cm alimentary limb (AL) before 2012, and the other way around (150 cm BPL and 70 cm AL) after 2012. OAGB was always performed with a BPL of 150 cm. In all conversion procedures, an intraoperative visualization of both crura of the diaphragm was done and a hiatoplasty was performed, if necessary.

### Gastroscopy

Gastroscopies prior to the SG as well as before the conversion were performed in all patients, except in acutely converted patients. For this study, all patients were asked to come in for a standardized gastroscopy at the Medical University of Vienna. Standardized biopsies were taken from the anastomosis, the pouch, and the gastroesophageal (GE) junction. The Seattle protocol (biopsies from every quadrant, every 1–2 cm starting at the ora serrata, and from suspicious mucosal areas at the distal esophagus) was applied in order to detect Barrett’s esophagus (BE) and reflux-related lesions at the GE junction [11].

### Esophageal Manometry and 24-h pH-Metry

Esophageal manometries and 24-h pH-metries were performed for the purpose of this study to evaluate the lower esophageal sphincter pressure (LESP) and the number and severity of acid and non-acid reflux activities within 24 h. This data was used to calculate the DeMeester score for each patient. These two examinations help to quantify the esophageal motility in the swallowing process as well as the severity of GERD.

### Quality of Life

Patients’ quality of life in the current study was evaluated using the Gastrointestinal Quality of Life Index (GIQLI) [12], which provides data on patients’ Gastrointestinal Quality of Life; the Bariatric Analysis and Reporting Outcome System (BAROS) [13] to assess the outcomes of a bariatric procedure in a long-term follow-up; and the Short Form (SF36) [14], which is a general quality-of-life score. Additionally, the Bariatric Quality of Life Index (BQL) [15] was assessed.

### Statistical Analysis

Data in this study are presented as median and range, mean and standard deviation, or as percentages (if appropriate). For the comparison of groups of data, *χ*^2^ tests and the nonparametric Mann–Whitney *U* test were used. Each univariate analysis was two-tailed; significance was set at a *p* value of < 0.05. Statistical calculations were conducted using SPSS V24 for Windows (IBM Corp., Armonk, NY).

## Results

Seventy-nine patients were converted from SG to another bariatric procedure between 01/2003 and 12/2018 at the Medical University of Vienna. Most patients were converted to RYGB (*n* = 54/68.4%), followed by OAGB (*n* = 17/21.5%) and SADI-S (*n* = 8/10.1%) (Table 1). The main reasons for the conversions were weight regain/insufficient weight loss (*n* = 44/55.7%), GERD (*n* = 27/34.2%), and acute conversions (*n* = 8/10.1%). In total, 48.1% (*n* = 38) of the patients were suffering from both weight regain/insufficient weight loss and GERD before the conversion (Table 1).

**Table 1:** Patient characteristics in patients converted from SG to RYGB, OAGB, and SADI-S (*n* = 79)

	***All patients (SG)***
	***n* = 79**
Sex (female) (*n* = 63)	79.7%
**Bariatric/reflux procedures before SG (*n* = 12)**	15.2%
Gastric banding (*n* = 9)***	11.4%
Gastric stimulation (*n* = 1)***	1.3%
Fundoplication (*n* = 2)***	2.5%
Gastric balloon (*n* = 2)	2.5%
**Converted patients (*n* = 79)**	
RYGB (*n* = 54)	68.4%
OAGB (*n* = 17)	21.5%
SADI-S (*n* = 8)***	10.1%
**Main indication for conversion**	
GERD (*n* = 27)*	34.2%
RYGB (*n* = 27)	
Weight regain (*n* = 44)*	55.7%
RYGB (*n* = 19)	
OAGB (*n* = 17)	
SADI-S (*n* = 8)***	
Others (dysphagia, leak, etc.) (*n* = 8)**	10.1%
RYGB (*n* = 8)	
Mean interval SG—conversion (in months)	58.7 ± 44.4
Follow-up after conversion (in months)	60.3 ± 41.0
Deceased patients (*n* = 1)***	1.3%

*Abbreviations: SG*, sleeve gastrectomy; *RYGB*, Roux-en-Y gastric bypass; *OAGB*, one-anastomosis gastric bypass; *SADI-S*, single-anastomosis duodeno-ileal bypass + sleeve gastrectomy; *R*, range.

*38 out of 79 (48.1%) patients were suffering from both weight regain and GERD.

**6 out of 8 (75.0%) patients were transferred from external hospitals to our bariatric center.

***Patients with bariatric/reflux procedures before SG and patients converted to SADI-S as well as one deceased patient were excluded from the analysis.

Sixty-seven patients had SG as a primary procedure, nine had gastric banding, one had gastric stimulation, and two had fundoplication before the SG. Additionally, two patients had endoscopic gastric balloon therapy before SG (Table 1). One patient deceased during the follow-up period of this study; however, the patient’s death was unrelated to the bariatric procedure and the conversion. Patients with bariatric/reflux procedures before SG (*n* = 12), patients converted to SADI-S (*n* = 8), and the deceased patient mentioned above were excluded from the analysis.

In total, 58 patients converted from primary SG to either RYGB or OAGB were analyzed in this study. Eight patients were converted in an acute setting within the first 30 days after SG, six of whom were transferred from external hospitals to our bariatric center. Reasons for these acute conversions were a leak at the most upper part of the staple line in five and a stenosis at the area of the incisura angularis in three patients. All of them were converted to RYGB. The mean time period between the SG and the conversion was 59.8 ± 45.1 months and the mean follow-up period after the conversion to RYGB and OAGB was 58.7 ± 43.7 months and 64.7 ± 22.8 months, respectively (Table 2).

**Table 2:** History of weight in patients converted from SG to RYGB and OAGB (*n* = 58)

	All patients* (n = *58)*	*Conversions*	
		RYGB (*n* = 45)	OAGB (*n* = 13)
SG (*n* = 58)			
Weight (kg)	141.7 ± 49.9	139.1 ± 27.6	150.4 ± 36.6
BMI (kg/m^2^)	49.3 ± 9.7	48.4 ± 8.6	52.3 ± 12.7
Nadir SG (*n* = 58)			
Weight (kg)	100.1 ± 26.4	96.2 ± 24.2	113.3 ± 30.0
BMI (kg/m^2^)	34.7 ± 8.4	33.5 ± 7.8	39.1 ± 9.0
Change BMI (kg/m^2^)	14.6 ± 6.2	14.9 ± 4.2	13.2 ± 8.3
EWL (%)	62.8 ± 30.8	67.3 ± 22.2	47.5 ± 32.3
TWL (%)	29.4 ± 13.3	30.9 ± 13.5	24.7 ± 13.2
Mean post-OP time (months)	18.9 ± 17.5	20.5 ± 19.1	13.8 ± 9.8
Conversion (*n* = 58)			
Weight (kg)	114.8 ± 28.3	110.0 ± 26.8	130.6 ± 28.2
BMI (kg/m^2^)	39.9 ± 8.8	38.6 ± 8.6	45.0 ± 7.3
Interval SG—conversion (months)	59.8 ± 45.1	60.5 ± 47.3	57.4 ± 38.3
Nadir conversion (*n* = 56)			
Weight (kg)	81.9 ± 22.4	81.4 ± 21.9	83.5 ± 24.7
BMI (kg/m^2^)	28.5 ± 7.1	28.4 ± 7.0	28.8 ± 7.7
Change BMI (kg/m^2^)**	20.8 ± 8.7	20.0 ± 5.7	23.5 ± 9.2
EWL (%)**	89.5 ± 29.0	89.0 ± 30.1	91.2 ± 26.1
TWL (%)**	42.2 ± 13.2	41.5 ± 14.1	44.8 ± 12.6
Median post-OP time (months)***	15.1 ± 14.3	14.9 ± 14.8	15.7 ± 6.4
Current (*n* = 55)			
Weight (kg)	87.8 ± 25.0	86.7 ± 25.1	91.0 ± 25.1
BMI (kg/m^2^)	30.6 ± 8.3	30.3 ± 8.5	31.4 ± 8.1
Change BMI (kg/m^2^)**	18.7 ± 7.9	18.1 ± 5.6	20.9 ± 9.3
EWL (%)**	79.9 ± 31.7	79.8 ± 34.1	80.3 ± 23.7
TWL (%)**	38.0 ± 13.1	37.7 ± 14.6	39.5 ± 11.5
Median post-OP time (months)***	60.0 ± 39.5	58.7 ± 43.7	64.7 ± 22.8

*Abbreviations: SG*, sleeve gastrectomy; *BMI*, body mass index; *EWL*, excess weight loss; *TWL*, total weight loss; *OP*, operation; *OAGB*, one-anastomosis gastric bypass; *RYGB*, Roux-en-Y gastric bypass

*Patients with bariatric/reflux procedures before SG and patients converted to SADI-S as well as one deceased patient were excluded from the analysis

**Referring to SG

***Referring to conversion

Data on the history of weight, undesirable symptoms, and complications were available in 55/58 (94.8%) patients. The follow-up rates for the examinations were 40/58 (68.9%) for gastroscopy, 21/58 (36.2%) for esophageal manometry, 21/58 (36.2%) for 24-h pH-metry, and 25/57 (43.1%) for the questionnaires.

### Weight Loss

The mean weight and BMI at the time of the SG were 141.7 ± 49.9 kg and 49.3 ± 9.7 kg/m^2^. The mean lowest postoperative weight and BMI in these patients were 100.1 ± 26.4 kg and 34.7 ± 8.4 kg/m^2^ after a mean period of 18.9 ± 17.5 months (total weight loss (TWL): 29.4 ± 13.3%). Weight and BMI at the time of the conversion were 114.8 ± 28.3 kg and 39.9 ± 8.8 kg/m^2^.

Patients converted to RYGB were able to reach a nadir weight and BMI of 81.4 ± 21.9 kg and 28.4 ± 7.0 kg/m^2^ at 14.9 ± 14.8 months after the conversion and 86.7 ± 25.1 kg and 30.3 ± 8.5 kg/m^2^ at the end of the follow-up (58.7 ± 43.7 months). There was no significant difference between patients converted before 2012 (*n* = 6) and after 2012 (*n* = 38). The group of patients converted to OAGB reached a weight and BMI of 83.5 ± 24.7 kg and 28.8 ± 7.7 kg/m^2^ at 15.7 ± 6.4 months after the conversion (nadir weight) and 91.0 ± 25.1 kg and 31.4 ± 8.1 kg/m^2^ at the end of the follow-up (64.7 ± 22.8 months). The TWL of patients converted to RYGB and OAGB, respectively, at nadir (Table 2). There were no significant differences between the converted groups in terms of weight loss (*p* = 0.46).

### Associated Medical Problems

The history of associated medical problems (AMP), i.e., arterial hypertension, diabetes mellitus type 2, hyperlipidemia, and obstructive sleep apnea, is listed in Table 3. Additionally, data on GERD are listed in this table as well. Between the SG and the conversion, the number of patients with AMP was relatively stable. The current number of patients with AMP is lower in all categories and for both conversion procedures. Exact numbers are listed in Table 3.

**Table 3:** Development of AMP and GERD after conversion from SG to RYGB and OAGB (*n* = 58)

	SG* (n = 58)	Conversion (n = 58)	Current	
			RYGB (n = 45)	OAGB (n = 13)
Arterial hypertension	23 (39.7%)	26 (44.1%)	9 (20.0%)	3 (23.0%)
Diabetes mellitus II	10 (17.2%)	10 (17.2%)	1 (2.2%)	2 (15.4%)
Hyperlipidemia	11 (19.0%)	8 (13.8%)	1 (2.2%)	2 (15.4%)
Obstructive sleep apnea	6 (10.3%)	5 (8.6%)	1 (2.2%)	1 (7.7%)
GERD	1 (1.7%)**	36 (62.0%)***	13 (28.9%)	7 (53.8%)

*Abbreviations: AMP*, associated medical problems; *SG*, sleeve gastrectomy; *OAGB*, one-anastomosis gastric bypass; *RYGB*, Roux-en-Y gastric bypass; *GERD*, gastroesophageal reflux disease

*Patients with bariatric/reflux procedures before SG and patients converted to SADI-S as well as one deceased patient were excluded from the analysis

**Despite GERD being a contraindication in our bariatric center, one patient insisted on SG as their bariatric procedure

***GERD was not the main indication for the conversion in all patients

### Peri-/Postoperative Complications

As mentioned earlier, eight patients were converted from SG to RYGB in acute settings. Three of them had a stenosis of the sleeve, which was resolved by the conversion. Five patients had leaks at the most upper part of the staple line of the sleeve and received further treatment with stenting or endoluminal VAC in addition to the conversion. All of these issues were resolved successfully.

In the rest of the collective (*n* = 36), postoperative complications were one stenosis of the anastomosis after OAGB, which was treated with balloon dilatation, and one wound infection of a trocar insertion that was treated conservatively.

### Reflux, Hiatal Hernias, and Barrett’s Esophagus

At the time of the SG, only one patient (1.7%) converted later was suffering from GERD. In the gastroscopies, performed preoperatively before each SG, none of the patients had any larger hiatal hernias. Despite the fact that GERD is a contraindication to SG at our bariatric center, this one patient insisted on having a SG as their bariatric procedure. In the evaluation before the conversion, 62.0% (*n* = 36) of the patients were suffering from GERD and 10.3% (*n* = 6) from short segment BE without dysplasia. These six patients with BE were converted to RYGB. In four of them, a complete remission of BE was observed. The current follow-up gastroscopies revealed two patients in the RYGB and one in the OABG group having developed short segment BE without dysplasia (Table 4).

**Table 4:** Endoscopic and histologic gastroscopy findings after conversion from SG to RYGB and OAGB (*n* = 40)

	**RYGB** (*n* = 31)	**OAGB** (*n* = 9)
**Macroscopic**		
Hiatal hernia	0 (0.0%)	0 (0.0%)
Bile in the pouch	0 (0.0%)	1 (11.1%)
Enlarged pouch*	2 (6.5%)	2 (22.2%)
CLE (GE junction)	5 (16.1%)	1 (11.1%)
CLE (length) in cm	1.8 (R 1–4)	1.0 (R 1–1)
**Microscopic**		
Esophagitis	4 (12.9%)	3 (33.3%)
Active anastomosis	5 (16.1%)	1 (11.1%)
Active pouchitis/gastritis of the sleeve	4 (12.9%)	1 (11.1%)
Barrett’s esophagus	4 (12.9%)**	1 (11.1%)
Helicobacter pylori	0 (0.0%)	1 (11.1%)

*Abbreviations: CLE*, columnar lined esophagus; *GE*, gastroesophageal; *OAGB*, one-anastomosis gastric bypass; *RYGB*, Roux-en-Y gastric bypass; *R*, range

*Enlarged sleeve was defined as possible inversion with a gastroscope equaling a 40 mm diameter.

**Two out of 4 patients had new onset of Barrett’s esophagus after conversion to RYGB; the other 2 patients had already had Barrett’s esophagus at the time of the conversion.

Currently, 29.9% (*n* = 13/45) are still symptomatic of GERD after RYGB and 53.8% (*n* = 7/13) after OAGB (Table 3). The histological examination of the current gastroscopies showed esophagitis in 12.9% and 33.3% after RYGB and OAGB and anastomosis in 16.1% and 11.1% of patients. Hiatal hernias were found in none of these patients. Further results of the gastroscopies performed after the conversions are highlighted in Table 4.

Results of the manometry were a mean LESP of 23.9 ± 11.8 mmHg after RYGB and 22.9 ± 7.4 mmHg after OAGB. There was no difference in the LESP of patients suffering from reflux compared to patients without reflux.

The 24-h pH-metry showed an acid reflux activity of 9.5 ± 16.2% and 0.7 ± 0.8% and a total number of refluxes in 24 h of 63.0 ± 33.4 and 84.3 ± 21.4 after RYGB and OAGB, respectively. The DeMeester score was calculated equaling 24.7 ± 27.8 and 3.3 ± 3.9 after RYGB and OAGB (Table 5).

**Table 5:** Esophageal manometry and 24-h pH-metry after conversion from SG to RYGB and OAGB (*n* = 21)

	**RYGB**	**OAGB**
**Manometry**	(*n* = 17)	(*n* = 4)
LESP (mmHg)		
(normal 10–35 mmHg)	23.9 ± 11.8	22.9 ± 7.4
Patients increased	4 (23.5%)	0 (0.0%)
Patients decreased	0 (0.0%)	0 (0.0%)
**24-h pH-metry**	(*n* = 17)	(*n* = 4)
Acid exposure (%)		
(normal < 4.2%)	9.5 ± 16.2	0.7 ± 0.8
Patients increased	7 (41.2%)	0 (0.0%)
Reflux activity (nr.)		
(normal < 73)	63.0 ± 33.4	84.3 ± 21.4
Patients increased	5 (29.4%)	2 (50%)
DeMeester score		
(normal < 14.72)	24.7 ± 27.8	3.3 ± 3.9
Patients increased	7 (41.2%)	0 (0.0%)

*Abbreviations: LESP*, lower esophageal sphincter pressure; *OAGB*, one-anastomosis gastric bypass; *RYGB*, Roux-en-Y gastric bypass

### Outcome and Quality of Life

In the Gastrointestinal Quality of Life Index (GIQLI), patients after RYGB and OAGB scored 93.0 ± 26.4 and 99.6 ± 18.1 points. The outcome of the bariatric procedures as expressed by BAROS was 3.6 ± 2.1 and 4.2 ± 0.8 after RYGB and OAGB. The BQL (a specific bariatric quality of life index) scores were 43.8 ± 12.9 for RYGB and 51.4 ± 10.0 for OAGB. The SF-36 questionnaire highlights the general quality of life (QOL) divided in four physical and four psychological categories. The SF-36 results for all 3 procedures are displayed in Table 6.

**Table 6:** Outcome scores and quality of life after conversion from SG to RYGB and OAGB (*n* = 25)

	RYGB *(n* = *20)*	OAGB *(n* = *5)*
GIQLI	93.0 ± 26.4	99.6 ± 18.1
BAROS	3.6 ± 2.1	4.2 ± 0.8
BQL	43.8 ± 12.9	51.4 ± 10.0
SF-36		
PF (physical functioning)	72.5 ± 24.3	80.6 ± 21.3
RP (role physical)	63.8 ± 43.1	68.7 ± 44.7
BP (bodily pain)	57.6 ± 30.1	71.0 ± 23.2
GH (general health)	57.2 ± 23.9	55.0 ± 21.1
VT (vitality)	44.2 ± 23.5	46.0 ± 21.1
SF (social functioning)	68.7 ± 27.5	81.0 ± 16.3
RE (role emotional)	70.4 ± 41.1	60.7 ± 47.1
MH (mental health)	58.4 ± 20.6	68.4 ± 14.0

*Abbreviations: SF36*, Short Form 36; *BQL*, Bariatric Quality of Life; *GIQLI*, Gastrointestinal Quality of Life Index; *BAROS*, Bariatric Analysis and Reporting Outcome System; *OAGB*, one-anastomosis gastric bypass; *RYGB*, Roux-en-Y gastric bypass

## Discussion

This study provides data from a mid-sized series of patients converted to RYGB and OAGB after failed SG. The study’s main findings are, first, a respectable amount of additional weight loss in both groups of patients converted from SG to either RYGB or OAGB. Second, conversions from SG to RYGB have improved GERD symptoms; however, not all patients are completely free of GERD symptoms today.

### Weight Regain, Additional Weight Loss, and Conversions

The decision which of the three procedures (RYGB, OAGB, SADI-S) our SG patients were converted to was made based on each individual patient. All patients had dietetic evaluation and gastroscopies (except acutely converted patients) before the conversion. Before 2015, all patients received either RYGB or OAGB, based on the most pressing issue amongst their symptoms. Thus, patients mainly suffering from GERD were mostly converted to RYGB, whereas patients with the priority symptom of weight regain/insufficient weight loss were converted to OAGB. All acutely converted patients had RYGB to create “dry” conditions (without any bile) in a low-pressure system. After 2015, patients suffering from weight regain/insufficient weight loss and without GERD have been converted to SADI-S.

In terms of perioperative morbidity, two complications occurred in the group of elective revisional procedures; however, both were resolved without reoperation. This outcome is within the benchmarks for elective secondary bariatric surgery recently defined by Gero D. et al. [16].

A recently published study with a follow-up of over 15 years after SG has shown that weight regain is a major issue in the long-term follow-up [17]. Therefore, any procedure a failed SG is converted to must have good potential to cause additional weight loss. For example, Rayman S. et al. compared a revision from SG to OAGB and RYGB in 119 and 144 patients, respectively, and found better weight loss but also higher rates of postoperative GERD in the OAGB group [18]. A study by Bashah M. et al. comparing SADI-S to OAGB as revisional procedures after SG for weight recidivism found better weight loss after SADI-S compared to OAGB and also better results in terms of GERD symptoms [19]. A meta-analysis by Matar R. et al. including 17 revisional studies converting patients from SG to RYGB found that RYGB induces a strong additional weight loss with a high resolution rate of GERD symptoms [20]. In the current study, the authors have found similar results in terms of weight loss, but OAGB has shown a lower rate of GERD remission than RYGB.

Of course, the amount of additional weight loss after a conversion from SG to another procedure not only depends on the procedure itself but majorly on the way it is done. Kraljevic M. et al. compared different biliopancreatic limb lengths in RYGB and OAGB as revisional procedures and found that a longer biliopancreatic limb should be considered in patients with weight regain and insufficient weight loss after SG [21].

Thus, major points when talking about revisional procedures are the length of the biliopancreatic and the common limb but also the question whether a re-shaping of the sleeve/pouch is done [22]. These potential differences make the studies and procedures in the literature hard to compare in terms of weight loss. Therefore, none of the revisional procedures (RYGB, OAGB) can in truth be considered inferior in terms of weight loss at this point.

### Associated Medical Problems

The improvement or remission of AMP is one of the most important results of any bariatric/metabolic procedure as well as for the patients’ individual life expectancies. In a study by Rayman S. et al. comparing OAGB to RYGB as revisional procedures after SG, both groups showed an improvement of AMP; however, there was no significant difference between both operations [18]. The current study found good additional remission rates after the conversion in all categories of AMP in both revisional procedures. Therefore, based on these findings, both revisional procedures may be considered equal in terms of AMP.

### GERD and Barrett’s Esophagus

Several recently published studies have shown that GERD and BE are major issues after SG [23, 24]. A study by Huynh D. et al. showed that after a conversion from SG to RYGB in 35 patients, the symptoms of GERD generally improved; however, there was still a subgroup of patients that were not able to discontinue proton pump inhibitors [25]. This finding is supported by the results of the present study as patients’ GERD symptoms did generally improve after the conversion to RYGB but unfortunately not all of them. This interesting fact shows that revisional RYGB may not be as effective in GERD remission as a primary RYGB, which is known as one of the best anti-reflux surgeries performed today [26]. This could be a result of an occurrence of hiatal hernias after SG. However, a visualization of both crura of the diaphragm was done intraoperatively in all patients of the current study at the time of the conversion and a hiatoplasty was performed in case any hiatal hernias were found. Interestingly, there was no difference in the LESP of this study’s patients with and without GERD, although one would guess that GERD patients might have had a lower LESP after revisional procedures.

A high complete remission rate of BE after SG has been reported after a conversion to RYGB [27]. In the current study, four out of six patients with short segment BE without dysplasia after SG had a complete remission after the conversion to RYGB; however, two additional cases of short segment BE without dysplasia were found as well. Of course, these findings might have been influenced by biopsy sampling errors that may occur when diagnosing short segment BE as described by Spechler and Souza in 2014 [28]. In any case, not only SG patients should have a surveillance gastroscopy every 5 years, but it should also be recommended for patients after a conversional procedure following a primary SG.

OAGB could be an option for GERD patients after SG as it provides a low-pressure system; however, if GERD is the prominent symptom and reason for the conversion, the pouch should be rendered as “dry” as possible. Saarinen T. et al. found bile in the pouch in up to 40% of the patients’ gastroscopies after OAGB [29].

Interestingly, 24-h pH-metry in this study revealed better outcomes for OAGB than for RYGB in terms of acid exposure and DeMeester score, even though more patients in the OAGB group were suffering from GERD symptoms. In fact, this may be a hint for the symptoms not being acid-based in these patients but triggered by biliary reflux. Gastroscopies can only provide a snapshot of biliary reflux, not a continuous measurement. Thus, further functional studies on this issue are certainly necessary to make any definitive statement. One may thus argue that RYGB still seems to be the best option when it comes to converting from SG in patients with severe GERD symptoms.

On the other hand, classic RYGB with a BPL of 70 cm and an AL of 150 cm might be less effective in terms of additional weight loss. Therefore, a possible solution may be extending the BPL to 150 cm in revisional patients as common in OAGB. This would combine the advantages of RYGB and OAGB in one procedure.

### Quality of Life

Evaluating patients’ QOL is one of the most important tools to measure the success of a bariatric/metabolic procedure. It is well-known that patients experience a significant improvement of their QOL after most bariatric procedures [30]. On the other hand, it has been shown that patients suffering from GERD or weight regain after SG have a decreased QOL compared to patients that do not suffer from any of these symptoms/changes [31]. There are hardly any studies describing patients’ QOL after revisional procedures following SG. There is only one study that compared primary to revisional single-anastomosis gastric bypass after restrictive procedures in 22 patients. The authors found a lower QOL after the revisional procedure [32]. This illustrates that QOL after revisional bariatric/metabolic surgery may be lower for the individual patient than after a primary procedure.

In the current study, patients after RYGB and OAGB reported almost equal QOL across all questionnaires; the results were only slightly better for OAGB. This is not surprising as both groups achieved proper weight loss and remission rates of AMP. On the other hand, there are patients with continuing GERD symptoms to be found in both groups, a symptom known to usually lower patients’ QOL. Furthermore, in the RYGB group, most of the patients initially had severe GERD after SG and the conversion did not completely solve the symptoms in all patients, as reported. In this group, the difference between the expectations and the actual results could be the reason for the slightly lower QOL in all scores.

## Strengths and Limitations of the Study

This study’s strengths are, first, a high follow-up rate in terms of patients’ history of weight. Second, it provides data from a further evaluation of patients using several objective examinations (gastroscopy, esophageal manometry, 24-h pH-metry, and several questionnaires) performed in our bariatric center specifically for the purpose of this study. Therefore, this study helps to evaluate two different conversion strategies by presenting the results of the abovementioned examinations.

However, the follow-up rates of the examinations were low. Specifically, the number of patients willing to undergo manometry and 24-h pH-metry was relatively low in the OAGB group so that individual results may have affected the outcome of these specific groups in an overproportioned way.

The number of patients converted to SADI-S was relatively low; therefore, these patients were excluded from this study. Another limitation was the fact that a small number of patients had gastric banding, gastric stimulation, or fundoplication before the SG, and were thus not included in this study, either.

Finally, in this retrospective study, the authors were not able to include any data from preoperative manometry and 24-h pH-metry examinations as they were not available from all patients.

## Conclusion

RYGB may be the best option when it comes to revisional procedures for patients with GERD after SG. In terms of additional weight loss and remissions of AMP, both procedures (RYGB and OAGB) were equal in this study’s population. OAGB has shown a low potential to cure patients from GERD symptoms after SG. In any case, surveillance gastroscopies every 5 years after SG revisions are recommended.
